# Unveiling Charge Transfer and Recombination Dynamics in 3D/2D Heterostructure via Ultrafast Spectroscopy for Efficient Perovskite Solar Cells

**DOI:** 10.1002/advs.202508123

**Published:** 2025-07-10

**Authors:** Di Li, Junhan Xie, Shaobing Xiong, Xiaoxiao Zang, Zhennan Lin, Yuning Wu, Weimin Liu, Bo Li, Zhenrong Sun, Junhao Chu, Qinye Bao

**Affiliations:** ^1^ School of Physics and Electronic Science Engineering Research Center for Nanophotonics and Advanced Instrument (MOE) East China Normal University Shanghai 200241 China; ^2^ School of Physical Science and Technology ShanghaiTech University Shanghai 201210 China; ^3^ Shanghai Frontiers Science Research Base of Intelligent Optoelectronics and Perception Institute of Optoelectronics Fudan University Shanghai 200433 China; ^4^ Collaborative Innovation Center of Extreme Optics Shanxi University Taiyuan Shanxi 030006 China

**Keywords:** 3D/2D perovskite heterostructure, charge transfer dynamics, nonradiative recombination, perovskite solar cell

## Abstract

Charge transfer properties between 3D and 2D perovskite layers play a key role in determining the performance of 3D/2D heterostructure perovskite solar cells (PSCs). However, the exact photophysical behaviors at 3D/2D perovskite heterostructure remain ambiguous, which makes it challenging to form the desired 3D/2D heterostructure. Herein, via combining the state‐of‐the‐art ultrafast spectroscopies of femtosecond transient absorption spectroscopy, transient absorption microscopy and time‐resolved photoluminescence spectroscopy, charge transfer and recombination dynamics are unveiled at 3D/2D perovskite heterostructure, for comparison, where the 2D layers are fabricated through the two distinct approaches of organic ligand surface reaction (2D_L_) and 2D crystal seed direct deposition (2D_S_), respectively. 3D/2D_S_ heterostructure exhibits superior hole transfer from 3D to 2D_S_, featuring a large spatial diffusion constant and high charge mobility compared to 3D/2D_L_, attributed to the higher phase purity and the lower defects in 2D_S_. Moreover, 3D/2D_S_ heterostructure yields suppressed nonradiative recombination, reduced Langevin recombination, and increased quasi‐Fermi level splitting, significantly aiding fast photoinduced charge transfer at such heterostructure. These advantages are further confirmed by a remarkably improved PSC efficiency using 3D/2D_S_, especially in terms of enhanced open‐circuit voltage and diminished energy loss. This work sheds light on the dynamics at 3D/2D heterostructures, providing a promising guideline for designing 3D/2D high‐performance PSCs.

## Graphical abstract

Charge transfer and recombination dynamics at 3D/2D perovskite heterostructure have been fully investigated via combined state‐of‐the‐art ultrafast spectroscopies, aiding to design of the desired 3D/2D perovskite heterostructure and thus push 3D/2D perovskite solar cell technology forward.

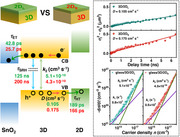

## 1 Introduction

Metal halide perovskite semiconductor has enabled remarkable advances in solar cells, light‐emitting diodes, photodetectors, and lasers, attributed to its superb optoelectronic properties of high light absorption coefficients, low exciton binding energies, long carrier diffusion lengths, and adjustable band gaps.^[^ ^1^, ^2^, ^3^, ^4^ ^]^ Despite notable progress of device efficiencies, the poor operational stability of perovskite optoelectronics is one main obstacle to achieving commercialization, due to the hydrophilic nature of organic components, low activation energy for ion migrations, and high defect densities at grain boundaries in commonly used polycrystalline 3D perovskite semiconductors.^[^ ^5^, ^6^, ^7^, ^8^ ^]^ Reduced‐dimensional perovskite semiconductors, e.g. 2D perovskite, have sparked great interest attributed to the enhanced stability from organic ligands as spacer molecules introduced into the perovskite structure, which increases moisture tolerance with suppressed ion migration.^[^ ^9^, ^10^ ^]^

3D/2D perovskite heterostructure is a promising strategy to combine the merits of the excellent photovoltaic efficiency of 3D perovskite and the high stability of 2D perovskite, leading to success, especially in perovskite solar cells (PSCs).^[^ ^11^, ^12^ ^]^ For 3D/2D perovskite heterostructure, the 2D perovskite is embedded within the grain boundaries of 3D perovskite matrix, efficiently passivating surface defects of 3D perovskite and improving the photovoltage of PSCs.^[^ ^13^, ^14^ ^]^ The 2D perovskite also blocks the channels of ion migration due to the relatively high energy barrier, which enhances the device stability and alleviates the current density‐voltage (*J*‐*V*) hysteresis.^[^ ^15^, ^16^ ^]^ In addition, it is witnessed that the introduction of 2D perovskite can form favorable interfacial energetics that accelerate charge extraction between 3D perovskite and the charge transport layer, thereby suppressing charge carrier recombination in devices.^[^ ^11^, ^17^, ^18^, ^19^ ^]^ To date, the 3D/2D perovskite heterostructures have been extensively employed in PSCs, helping to significantly increase the device efficiency and stability.^[^ ^20^, ^21^ ^]^ Nevertheless, the exact ultrafast charge transfer and recombination dynamics at 3D/2D perovskite heterostructure are still ambiguous, which seriously limits to construct the desired 3D/2D heterostructure and pushes the 3D/2D PSC technology forward.

In this work, we combine complementary transient spectroscopies and comparable device performances to fully investigate charge transfer and recombination dynamics in 3D/2D perovskite heterostructures for n‐i‐p PSCs, in which the 2D perovskite layers are grown using the two distinct approaches of organic ligand surface reaction (2D_L_) and 2D crystal seed direct deposition (2D_S_), respectively. By adopting femtosecond transient absorption (fs‐TA) spectroscopy, we find that 3D/2D_S_ heterostructure yields superior electron transfer process across from 3D/2D_S_ to the electron transfer layer compared to 3D/2D_L_ heterostructure, which is mainly attributed to the higher phase purity and the lower defects in the 2D_S_. Transient absorption microscopies (TAM) reveal that 3D/2D_S_ heterostructure exhibits a larger spatial diffusion constant (0.175 cm^2^ s^−1^) and a higher charge mobility (6.8 cm^2^ V^−1^ s^−1^) than 3D/2D_L_ heterostructure (0.105 cm^2^ s^−1^; 4.1 cm^2^ V^−1^ s^−1^). Moreover, time‐resolved photoluminescence (TRPL) measurements prove that 3D/2D_S_ heterostructure features suppressed nonradiative recombination, reduced Langevin recombination, and enlarged quasi‐Fermi level splitting. These advances in dynamics are further verified by the greatly improved PSC performance with 3D/2D_S_ heterostructure, especially in terms of enhanced open‐circuit voltage (*V*_oc_) and fill factor (FF), as well as reduced energy loss, compared to the device with 3D/2D_L_ heterostructure. In addition, we achieved one of the highest efficiencies of 22.32% and *V*_oc_ of 1.151 V of 3D/2D heterostructure MAPbI_3_ PSCs reported to date. By unveiling the photophysical mechanism at the heterostructure, this work provides a guideline for designing effective 3D/2D perovskite heterostructure to enable high‐performance PSCs.

## 2 Results and Discussion

**Figure 1:**
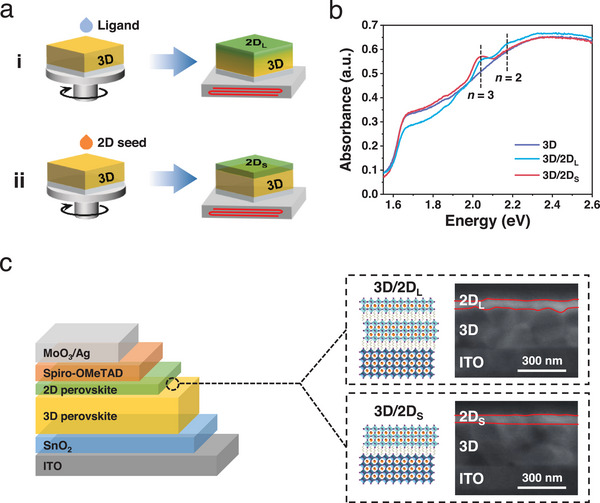
(a) Schematics of 3D/2D_L_ and 3D/2D_S_ perovskite heterostructure fabrication processes. (b) UV–vis absorption spectra of bare 3D, 3D/2D_L,_ and 3D/2D_S_ heterostructure films. (c) Device structure of n‐i‐p PSCs employing 3D/2D_L_ and 3D/2D_S_ as light absorbers, respectively. The insets represent schematic illustrations and cross‐sectional SEM images of 3D/2D_L_ and 3D/2D_S_ heterostructures, respectively.

**Figure** **1**a illustrates the two distinct routes for fabricating 3D/2D perovskite heterostructures. Here, the MAPbI_3_ serves as 3D perovskite in this study. In route (i), the isopropyl alcohol (IPA) containing iodized organic ligands n‐butylamine iodide (BAI) is spin‐coated on the as‐prepared 3D MAPbI_3_ perovskite film, followed by a post‐thermal treatment process, where the 2D perovskite (referred as 2D_L_) gradually grows via in situ cation exchange and reaction with excess PbI_2_ of 3D perovskite to form a 2D_L_ layer capping on the 3D film.^[^ ^22^ ^]^ On the contrary, in route (ii), the 2D crystal seed solution (BA_2_MA_2_Pb_3_I_10_) is directly deposited on the top of the 3D perovskite MAPbI_3_ film, forming a 2D perovskite layer (referred as 2D_S_). The acetonitrile solvent with an appropriate dielectric constant and donor strength avoids the consumption of the underlying 3D perovskite.^[^ ^11^ ^]^ The route (ii) is expected to produce the 2D_S_ perovskite with the higher phase purity compared to the route (i), as demonstrated by the UV–visible absorption spectra (Figure 1b). The 3D/2D_L_ perovskite heterostructure shows two excitonic peaks of 2.17 and 2.04 eV, indicating the mixed phases of 2D perovskite (BA_2_MA*_n_*_‐1_Pb*_n_* I_3_ *_n_*_+1_, *n* = 2 and *n* = 3), while the 3D/2D_S_ perovskite heterostructure delivers the pure phase of 2D perovskite (*n* = 3) with a single excitonic peaks of 2.04 eV. In addition, we find that the 3D/2D_S_ heterostructure has a smoother heterointerface between 3D and 2D_S_ than that of 3D/2D_L_ heterostructure due to the reaction at the heterointerface, as observed in the cross‐sectional scanning electron microscopy (SEM) images of 3D/2D_L_ and 3D/2D_S_ perovskite heterostructures (Figure 1c). Figure 1c also shows the device structure of the n‐i‐p PSCs employing 3D/2D_L_ and 3D/2D_S_ heterostructures for comparison in this work.

**Figure 2:**
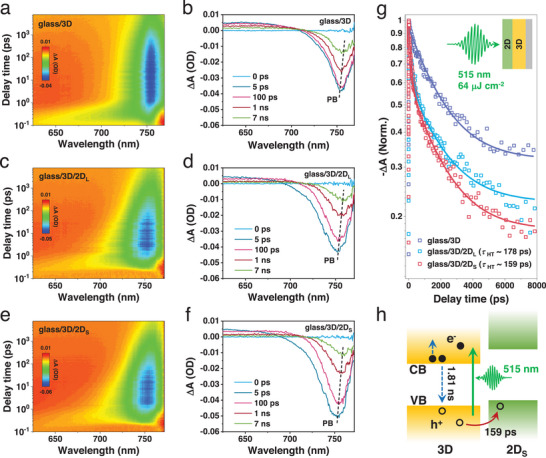
fs‐TA spectra and different probe delay times of (a,b) bare 3D, (c,d) 3D/2D_L,_ and (d,e) 3D/2D_S_ heterostructures. (g) Decay kinetics of bare 3D, 3D/2D_L_ and 3D/2D_S_ heterostructures. (h) hole transfer dynamics at 3D/2D_S_ heterointerface.

The fs‐TA spectroscopy provides a robust tool for monitoring interfacial carrier transfer dynamics at the 3D/2D perovskite heterostructure,^[^ ^23^, ^24^, ^25^, ^26^ ^]^ where the measurements are performed under photoexcitation at 515 nm with a pump fluence ≈64 µJ cm^−2^. The bare 3D perovskite, 3D/2D_L,_ and 3D/2D_S_ heterostructures are deposited on the insulated glasses to avoid interference from the underlying conductive electrode. **Figure** **2**a–f shows the pseudo‐color fs‐TA maps and corresponding extracted spectra at various delay times of 3D perovskite, 3D/2D_L,_ and 3D/2D_S_ heterostructures. The most pronounced photobleaching (PB) peaks in the three samples all exhibit a blueshift from 758 to 750 nm within the initial 600 fs and then a redshift back to 758 nm within 7 ns (dotted lines), attributed to the competition between the Burstein‐Moss effect causing the band gap to the higher energy and band gap renormalization (BGR) causing the band gap narrowing.^[^ ^27^, ^28^ ^]^ We plot their normalized PB decay traces fitted by the multi‐exponential decay equation:^[^ ^29^, ^30^, ^31^ ^]^ $y=\sum_{i=1,2}A_{i}\exp\left(-x/\tau_{i}\right)\;+y_{0}$ (Figure 2g), where τ_*i*_ is the process lifetime, *A_i_* is corresponding amplitude, *i* represents mono‐exponential decay or bi‐exponential decay, respectively, and *y*_0_ is offset. Table S1 (Supporting Information) summarizes the fitted parameters of the charge transfer dynamics. All three samples show a similar slow decay component that originates from the Auger recombination process in 3D bulk perovskite with *τ*_Auger_ ∼1.84 ± 0.04 ns.^[^ ^32^ ^]^ A long, infinite lifetime component *τ*_long_ is limited by the time window, which will be discussed in the later TRPL measurements. Compared to the bare 3D perovskite, we find an additional fast decay component of hole transfer (HT) in the heterostructures, which yields smaller lifetime *τ*_HT_ of ≈159 ps at the 3D/2D_S_ heterostructure than ≈178 ps at the 3D/2D_L_ heterostructure, indicating that the hole transfer occurs between 3D and 2D across the heterointerface,^[^ ^19^ ^]^ and the 3D/2D_S_ heterostructure features more efficient hole transfer than the 3D/2D_L_ heterostructure (Figure 2h).^[^ ^11^, ^33^ ^]^

**Figure 3:**
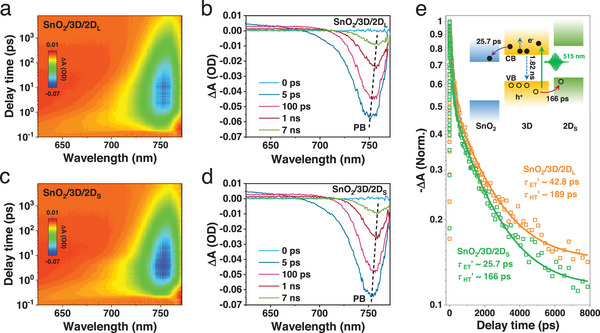
fs‐TA spectra and different probe delay times of (a,b) 3D/2D_L_ and (c,d) 3D/2D_S_ films on SnO_2_ for n‐i‐p PSCs. (e) Decay kinetics of SnO_2_/3D/2D_L_ and SnO_2_/3D/2D_S_ heterostructures. The insert illustrates electron and hole transfer dynamics at SnO_2_/3D/2D_S_ heterointerfaces.

To gain insights into the impact of the 3D/2D heterostructure on the carrier transfer in the device, we measure the pseudo‐color fs‐TA spectra of the half‐stacked devices SnO_2_/3D/2D_L_ and SnO_2_/3D/2D_S_ with electron transport layer (ETL) for n‐i‐p architecture PSCs, and the transient spectra at various delay times are displayed in **Figure** **3**a–d. The PB signals also present a redshift due to the competition between Burstein‐Moss effect and BGR. As shown in Figure 3e and Table S2 (Supporting Information), the SnO_2_/3D/2D_S_ heterostructure exhibits enhanced interfacial hole transfer (τ_HT_′ ≈ 166 ps) compared to the SnO_2_/3D/2D_L_ (τ_HT_′ ≈ 189 ps), which agree well with the results of 3D/2D_S_ and 3D/2D_L_ heterostructure without ETL^[^ ^34^ ^]^ (Figure 2). When introducing the SnO_2_ ETL, a decay component of the electron transfer (ET) from 3D perovskite to SnO_2_ emerges, which is obviously faster in the SnO_2_/3D/2D_S_ (τ_ET_′ ≈ 25.7 ps) than in the SnO_2_/3D/2D_L_ (τ_ET_′ ≈ 42.8 ps). The SnO_2_/3D/2D_S_ has a higher amplitude ≈13.5% of the interfacial electron transfer than ≈12.4% in the SnO_2_/3D/2D_L_. The reason is that more efficient hole transfer from 3D to 2D_S_ perovskite decreases electron attraction ability within 3D perovskite, which would effectively promote electron transfer from 3D to adjacent SnO_2_ ETL across the interface.^[^ ^35^ ^]^ We thus conclude that the SnO_2_/3D/2D_S_ has both excellent electron and hole transfer efficiencies compared to the SnO_2_/3D/2D_L_.

**Figure 4:**
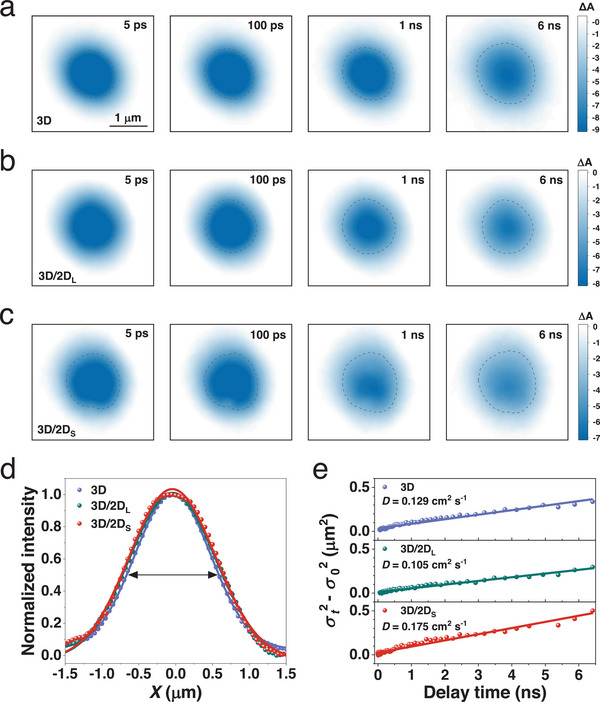
TAM images at 750 nm of (a) bare 3D, (b) 3D/2D_L,_ and (c) 3D/2D_S_ heterostructures as a function of delay times, respectively. The dashed circle means FWHM. (d) 1D TAM profiles with delay time at 5 ps fitted by Gaussian function. (e) Diffusion coefficients of bare 3D, 3D/2D_L_ and 3D/2D_S_ heterostructures.

We employ the TAM measurements to monitor the transient temporal and spatial response of charge carrier behavior at the 3D/2D perovskite heterostructure. **Figure** **4**a–c shows the TAM images captured at various delay times for bare 3D, 3D/2D_L,_ and 3D/2D_S_ heterostructures, respectively. The dashed circles represent the full width at half maximum (FWHM) of the signals, and the magnifies of the circles as time delay means carrier diffusion. The TAM profiles of bare 3D, 3D/2D_L,_ and 3D/2D_S_ at 5 ps are well fitted by a 1D Gaussian function in Figure 4d (more details see the Note S1, Supporting Information). Figure 4e illustrates the variation of the photogenerated carrier density profile $\sigma_{t}^{2}-\sigma_{0}^{2}$ as a function of delay time. A strong linear dependence gives the spatial diffusion constant *D* of 0.129 cm^2^ s^−1^ for the bare 3D, 0.105 cm^2^ s^−1^ for the 3D/2D_L_ heterostructure, and 0.175 cm^2^ s^−1^ for the 3D/2D_S_ heterostructure, respectively. The smallest *D* of the 3D/2D_L_ heterostructure is ascribed to the most defects induced by the mixed 2D phases. The highest *D* of the 3D/2D_S_ heterostructure originates from the defect‐passivated 3D/2D_S_ heterointerface and the pure 2D phases, which are expected to yield excellent device performance. Moreover, we calculate the carrier mobility *μ* following the Einstein relation:^[^ ^36^ ^]^ $\frac{D}{\mu }=\frac{kT}{q}\;$, where *k* is Boltzmann constant, *T* is temperature, and *q* is carrier charge. The *μ* values are estimated to be 5.0 cm^2^ V^−1^ s^−1^ for the bare 3D, 4.1 cm^2^ V^−1^ s^−1^ for the 3D/2D_L_, and 6.8 cm^2^ V^−1^ s^−1^ for the 3D/2D_S_, respectively. The 3D/2D_S_ heterostructure has the highest carrier mobility which leads to the best FF in PSCs.

**Figure 5:**
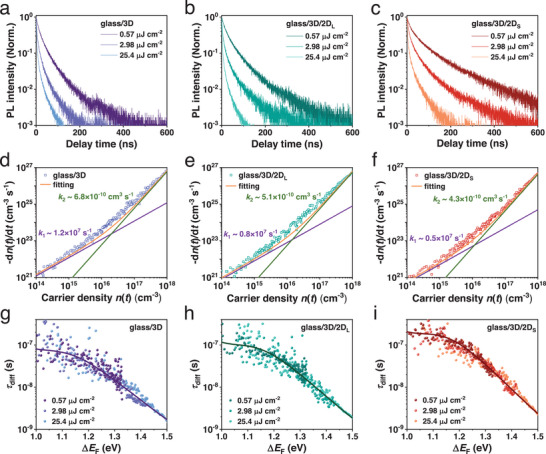
TRPL spectra of (a) bare 3D, (b) 3D/2D_L,_ and (c) 3D/2D_S_ heterostructures with different pump fluences. Recombination rate ‐d*n*(*t*)/d*t* over charge density *n*(*t*) for (d) bare 3D, (e) 3D/2D_L_ and (f) 3D/2D_S_. The purple and green lines indicate the contributions from monomolecular and bimolecular recombination, respectively. Differential decay time as a function of quasi‐Fermi level splitting for (g) bare 3D, (h) 3D/2D_L,_ and (i) 3D/2D_S_, where the curves are guidance to the eye.

To explore the charge recombination dynamics at the 3D/2D perovskite heterostructure, we carefully perform the PL and TRPL measurements. The steady‐state PL spectra of 3D/2D_L_ and 3D/2D_S_ heterostructures exhibit enhanced intensity and slightly broadened emission compared to the bare 3D film (Figures S1–S3, Supporting Information). In the TRPL (**Figure** **5**a–c), the 3D/2D_L_ and 3D/2D_S_ heterostructures also have prolonged carrier recombination lifetimes. To elucidate the competitive recombination mechanisms of the photoinduced carriers, the relaxation kinetics are described by the recombination rate over the whole carrier density.^[^ ^37^ ^]^ We plot the time derivatives of the carrier density d*n*(*t*)/d*t* with respect to the carrier density *n*(*t*) in Figure 5d–f. The recombination rate displays a linear dependence on *n*(*t*) at low carrier density and then transits to a quadratic dependence at high carrier density. The kinetics are modeled via a quadratic equation:^[^ ^38^, ^39^ ^]^ $-\;\frac{dn(t)}{dt}=k_{2}\;n{\left(t\right)}^{2}+k_{1}n\left(t\right),$where *k*_1_ = (*τ*_SRH_)^−1^ is the monomolecular defect‐assisted recombination coefficient, *τ*_SRH_ is the defect‐assisted recombination (i.e. Shockley‐Read‐Hall recombination) lifetime, and *k*_2_ is the bimolecular radiative recombination coefficient, respectively. As listed in **Table** **1**, the obtained defect‐assisted monomolecular recombination coefficients *k*_1_ are 1.2 × 10^7^ s^−1^ for the bare 3D, 0.8 × 10^7^ s^−1^ for the 3D/2D_L_ and 0.5 × 10^7^ s^−1^ for the 3D/2D_S_, respectively, which correspond to the *τ*_SRH_ values of 83 ns, 125 ns, and 200 ns, respectively. The longest *τ*_SRH_ in the 3D/2D_S_ heterostructure implies the smallest defect densities and minimized nonradiative recombination.^[^ ^40^ ^]^

**Table 1:** Carrier decay constant, mobility, and Langevin ratio of bare 3D, 3D/2D_L,_ and 3D/2D_S_ heterostructures for comparison.

	Carrier decay constant		Mobility	Langevin ratio
	*k*_2_ [cm^3^ s^−1^]	*τ*_SRH_ (ns)	*μ* ([cm^3^ V^−1^ s^−1^]	*δ*
3D	6.8 × 10^−10^	83	5.0	7.5 × 10^−5^
3D/2D_L_	5.1 × 10^−10^	125	4.1	6.8 × 10^−5^
3D/2D_S_	4.3 × 10^−10^	200	6.8	3.5 × 10^−5^

The bimolecular radiative recombination coefficient *k*_2_ values are calculated to be 6.8 × 10^−10^ cm^3^ s^−1^ for the bare 3D, 5.1 × 10^−10^ cm^3^ s^−1^ for the 3D/2D_L_ and 4.3 × 10^−10^ cm^3^ s^−1^ for the 3D/2D_S_, respectively. The bimolecular radiative recombination is associated with the Langevin model, describing that the Coulomb attraction predominates in materials in which the charge Coulomb radius is much larger than the mean free path, and the recombination occurs once electrons and holes move within the joint capture radius.^[^ ^41^ ^]^ *k*_2_ follows the equation:^[^ ^42^ ^]^ (*k*_2_/*μ*)_L_ = *q*/(*ε*_0_ *ε*_r_), where *ε*_0_ and *ε*_r_ are the permittivity of vacuum and material, respectively. Through defining the Langevin ratio (*δ*):^[^ ^42^, ^43^ ^]^ $\delta =\frac{{\left(k_{2}/\mu \right)}_{\exp}}{{\left(k_{2}/\mu \right)}_{L}}\;\varepsilon_{r}^{-1}$, the *δ* of 3.5 × 10^−5^ for the 3D/2D_S_ is smaller than the 7.5 × 10^−5^ for the bare 3D and 6.8 × 10^−5^ for the 3D/2D_L_ (Table 1), implying reduced Langevin recombination in the 3D/2D_S_ heterostructure. Additionally, the 3D/2D_S_ heterostructure has a broadened PL emission (Figures S1–S3, Supporting Information). The largest space charge separation leads to the reduced spatial overlap of electron and hole wave function and carrier recombination. The 3D/2D_S_ heterostructure produces the smallest Langevin ratio *δ* and hence reduces bimolecular radiative recombination rates without sacrificing carrier mobility.^[^ ^42^ ^]^

The quasi‐Fermi level splitting (QFLS, Δ*E*_F_) dominates the *V*_oc_ of the PSCs.^[^ ^44^ ^]^ After the laser pulse arrives, the concentration gradient of excited electrons and holes causes QFLS, which can be expressed as $\Delta E_{F}=kTln\left(\frac{\Delta n{\left(t\right)}^{2}}{n_{i}^{2}}\right)$,^[^ ^45^ ^]^ where *k* is the Boltzmann constant, *T* is the temperature, and Δ*n*(*t*) is free carrier density, respectively. *n*_i_ represents the intrinsic carrier density expressed as $n_{i}=\sqrt{N_{C}N_{V}}\;*exp\left(-\frac{E_{g}}{2k_{B}T}\right)$, where *E*_g_ is bandgap, $N_{C}=2{\left(\frac{2\pi m_{e}^{*}k_{B}T}{\hbar^{2}}\right)}^{3/2}$ and $N_{V}=2{\left(\frac{2\pi m_{h}^{*}k_{B}T}{\hbar^{2}}\right)}^{3/2}$ are effective density of states for conduction and valance band, ℏ is Planck constant, $m_{e}^{*}$ and $m_{h}^{*}$ are electron and hole effective mass, respectively. By defining the differential lifetime as $\tau_{\mathrm{diff}}={\left(-\frac{dln(I_{\mathrm{TRPL}})}{dt}\right)}^{-1}\;$,^[^ ^45^ ^]^ where *I*_TRPL_ is PL intensity, and the relevant recombination mechanisms are clearly distinguished via plotting *τ*_diff_ as a function of Δ*E*_F_. The differential lifetime *τ*_diff_ as a function of delay time is shown in Figures S4–S6 (Supporting Information). Due to bimolecular recombination, *τ*_diff_ increases at the early delay time and becomes more pronounced at the high pump fluence. The differential lifetime continues to increase until the monomolecular recombination works, and the *τ*_diff_ of the different pump power densities reaches a fixed lifetime *τ*_SRH_ (dashed lines) at the longer time. We plot the differential time *τ*_diff_ as a function of Δ*E*_F_ in Figure 5g–i. The bimolecular recombination determines the initial Δ*E*_F_ at the early time, where the 3D/2D_S_ heterostructure shows the larger Δ*E*_F_ compared to the 3D/2D_L_ heterostructure. It is ascribed to the smaller bimolecular recombination rate *k*_2_. At the longer time, the monomolecular recombination dominates the saturation of Δ*E*_F_ to *τ*_SRH_, and the 3D/2D_S_ heterostructure also features the larger Δ*E*_F_ due to the lower defect density at the heterostructure.

**Figure 6:**
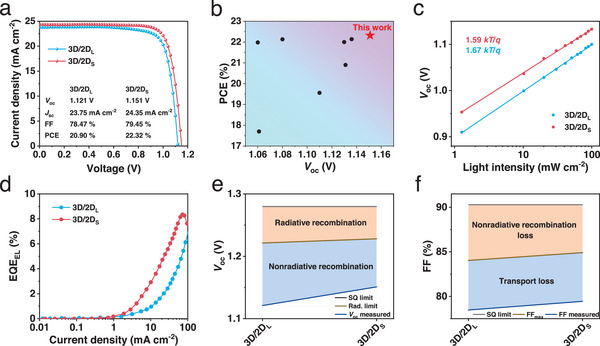
(a) *J*‐*V* curves of n‐i‐p PSCs employing 3D/2D_L_ and 3D/2D_S_ heterostructures. (b) The collection of PCE with respect to *V*_oc_ for recently reported 3D/2D heterostructure MAPbI_3_ PSCs. (c) Dependence of *V*_oc_ on light intensity for devices with 3D/2D_L_ and 3D/2D_S_ heterostructures. (d) EQE_EL_ spectra as a function of current density for devices with 3D/2D_L_ and 3D/2D_S_ heterostructures. (e) *V*_oc_ loss analysis and (f) FF loss analysis of devices with 3D/2D_L_ and 3D/2D_S_ heterostructures.

To further substantiate the dynamics advantages of the 3D/2D_S_ heterostructure in enhancing device performance, we fabricate the n‐i‐p MAPbI_3_ PSCs with a configuration of ITO/SnO_2_/3D/2D/Spiro‐OMeTAD/MoO_3_/Ag. **Figures** **6**a and S7 (Supporting Information) show the *J*‐*V* curves of the 3D/2D_L_ and 3D/2D_S_ heterostructure PSCs under AM1.5G simulated solar illumination. The device with 3D/2D_L_ heterostructure shows a power conversion efficiency (PCE) of 20.90%, with a *V*_oc_ of 1.121 V, a *J*_sc_ of 23.75 mA cm^−2^ and an FF of 78.47%, which are comparable with MAPbI_3_ PSCs. When employing 3D/2D_S_ heterostructure, the resulting device exhibits a remarkably improved PCE of 22.32%, with a higher *V*_oc_ of 1.151 V, *J*_sc_ of 24.35 mA cm^−2^ and FF of 79.45%, due to the enhanced charge transfer, large extensive carrier spatial diffusion, suppressed recombination and increased quasi‐Fermi level splitting at the 3D/2D_S_ heterostructure. To the best of our knowledge, the achieved PCE and *V*_oc_ are among the highest values for the 3D/2D heterostructure MAPbI_3_ PSCs reported in the literature (Figure 6b; Table S3, Supporting Information). The resulting 3D/2D_S_ heterostructure PSC shows negligible hysteresis (Figures S8 and S9, Supporting Information). The corresponding external quantum efficiency (EQE) spectrums yield integrated photocurrent densities in accordance with the *J*‐*V* scanning curves (Figure S10, Supporting Information). According to the equation:^[^ ^46^ ^]^ $V_{\mathrm{oc}}=\frac{nkTln(P)}{q}\;$, where *n* is ideality factor, *k* is Boltzmann constant, and *T* is absolute temperature, respectively, the resulting device with 3D/2D_S_ shows a smaller slope of 1.59 in terms of *kT*/*q* than the 1.67 slope of the device with 3D/2D_L_ (Figure 6c), suggesting the reduced defect‐assisted recombination due to the pure 2D_S_ phase. Moreover, as shown in Figure 6d, the external quantum efficiency of electroluminescence (EQE_EL_) value of the device largely increases from 2.11% (3D/2D_L_) to 5.15% (3D/2D_S_), pointing to the impressively diminished nonradiative recombination induced *qV*_oc_ loss from 100 to 77 meV as per the detailed balance theory:^[^ ^47^ ^]^ $\Delta V_{\mathrm{oc}}=\frac{kT}{q}\;\ln\left(\mathrm{EQ}E_{\mathrm{EL}}\right)$, where *k* is Boltzmann constant, *T* is absolute temperature and *q* is carrier charge, in consistent with the analysis of charge recombination dynamics (Figure 6e). The increased exponential factor *α* derived from *J*_sc_∝*P* ^α^,^[^ ^2^ ^]^ indicates the suppression of the interfacial charge recombination at the 3D/2D_S_ heterostructure (Figure S11, Supporting Information). The device maximum FF (FF_max_) without charge transport loss are estimated via the equation:^[^ ^48^ ^]^ $FF_{\max}=\frac{V_{A}-\ln\left(V_{A}+0.72\right)}{V_{A}+1}\;$, where$\;V_{A}=\frac{qV_{oc}}{nk_{B}T}\;$. The discrepancy of FF_max_ follows the suppression of nonradiative recombination loss in PSCs. The charge transport induced FF loss diminishes from 5.57% to 5.46% due to the increased charge transfer dynamics at the 3D/2D_S_ heterostructure (Figure 6f).

## 3 Conclusion

In summary, we have systemically explored the charge transfer and recombination dynamics of 3D/2D perovskite heterostructure for PSCs via a series of advanced ultrafast spectroscopies, including fs‐TA, TAM, and TRPL measurements. For comparison, the 2D perovskite capping layers are fabricated using the two distinct approaches of organic ligand surface reaction (2D_L_) and 2D crystal seed direct deposition (2D_S_), respectively. We have demonstrated that 3D/2D_S_ heterostructure yields superior charge transfer process across from 3D to 2D_S_, large spatial diffusion constant, high charge mobility, suppressed nonradiative recombination, reduced Langevin recombination, and increased quasi‐Fermi level splitting compared to 3D/2D_L_ heterostructure, which significantly aids fast photoinduced charge‐transfer at such heterostructure. Moreover, these advantages are further confirmed by a remarkably improved PSC efficiency with 3D/2D_S_ heterostructure, especially in terms of *V*_oc_, FF, and energy loss. Additionally, we have achieved one of the highest efficiencies of 22.32% and a *V*_oc_ of 1.151 of 3D/2D heterostructure MAPbI_3_ PSCs reported to date. This work provides a guideline for promoting the photophysical charge transfer process at 3D/2D heterostructure to improve PSC efficiency and stability.

## 4 Experimental Section

### Materials

Tin oxide (SnO_2_) colloid precursor was purchased from J&K Scientific. Lead iodide (PbI_2_), n‐butylamine iodide (BAI), 2,2',7,7'‐Tetrakis[N,N‐di(4‐methoxyphenyl)amino]‐9,9'‐spirobifluorene (Spiro‐OMeTAD), bis(trifluoromethanesulfonyl)imide lithium (Li‐TFSI) and tributyl phosphate (TBP) were received from Polymer Light Technology. Methylammonium iodide (MAI) was purchased from Advanced Election Technology Co. Ltd. 2D perovskite crystal powers (BA_2_MA_2_Pb_3_I_10_), N, N‐dimethylformamide (DMF), dimethyl sulfoxide (DMSO), chlorobenzene (CB), isopropyl alcohol (IPA) and acetonitrile (MeCN) were obtained from Sigma‐Aldrich.

### Ultrafast Femtosecond Transient Absorption Spectroscopy

fs‐TA spectroscopy was carried out by a commercial Ti: Sapphire laser system (Coherent, Astrella, 35 fs, 1 kHz repetition rate), which provides 800 nm fundamental beam. The pump beam at 515 nm was generated using an optical parametric amplifier (OPerA Solo, Coherent Inc.), which focuses on the samples with excitation fluence ∼64 µJ cm^−2^. The probe pulse served by the broadband supercontinuum white light with a wavelength of 630–770 nm. All tests were measured at room temperature.

### Transient Absorption Microscopy

TAM measurements were performed using 1030 nm laser generated from a commercial Fiber laser (100 kHz, < 290 fs, YactoFiber). 515 nm pump beam was produced by BBO crystal via second harmonic generation, which focuses on the samples with a microscope objective (×100, NA = 0.5, Olympus). 750 nm probe beam by a white light continuum was produced by a sapphire plate. The transmitted signals were collected by a charged metal‐oxide‐semiconductor (CMOS) detector (360 × 480 pixels, Basler acA640‐750 µm).

### Photoluminescence Spectroscopy

The picosecond laser (SC Pro 7, Wuhan Yangtze Soton Laser Co., Ltd.) with an excitation wavelength center at 515 nm was used to excite all samples, the repetition rate was 1 MHz and the excitation fluence was 0.57∼25.4 µJ cm^−2^. The instrument response function was 200 ps. The steady‐state PL spectroscopy was recorded by a charge‐coupled device (CCD), the spectrometer used here was iHR550 from HORIBA. The TRPL was recorded by an avalanche photodiode (APD) with time‐correlated single photon counting (TCSPC) technique (PicoHarp 300, PicoQuant). All measurements were conducted at room temperature.

### Device Fabrication and Characterization

The pre‐patterned ITO glasses were cleaned with detergent, deionized water, ethanol, and isopropanol for 20 mins in sequence, and then were treated with UV‐ozone for 20 mins. The SnO_2_ colloid precursor diluted with deionized water (1/6 v/v) was spin‐coated onto the ITO substrates at 4000 rpm for 30 s, and then annealed at 150 °C for 30 min. The 3D perovskite MAPbI_3_ solution was composed of 159.0 mg MAI and 497.8 mg PbI_2_ dissolving in the mixed solvent with 750 µL DMF and 85 µL DMSO, and the 3D perovskite was deposited on SnO_2_ at 4000 rpm for 30 s, 120 µL CB as antisolvent was dropped onto the film after 7 s processing, and then annealed at 100 °C for 10 min. To fabricate the 3D/2D perovskite heterostructure, the BAI ligand salt solution in IPA with a concentration of 5 mg mL^−1^ was deposited on the 3D perovskite at 4000 rpm for 30 s, and then the film was annealed at 100 °C for 5 min to form 2D_L_ perovskite. Otherwise, the seed solution of crystal powers (BA_2_MA_2_Pb_3_I_10_) in MeCN with a concentration of 10 mg/mL was deposited on 3D perovskite at 2000 rpm for 30 s, and then annealed at 85 °C for 5 min to form 2D_S_ perovskite. The upper hole transport layer Spiro‐OMeTAD (72.3 mg Spiro‐OMeTAD in CB with 28.8 µL TBP and 17.5 µL Li‐TFSI solution with a concentration of 520 mg/mL in acetonitrile) was deposited on the perovskite film via spin‐coating at 4000 rpm for 30 s. After storage in dry air overnight, 5 nm MoO_3_ and 100 nm Ag were thermally evaporated as the electrode.

The device current density‐voltage (*J*‐*V*) curves were collected under Enli solar simulator (SSF5‐3A, Enlitech) via a Keithley 2400 series source meter in N_2_‐filled glovebox without any preconditioning, and the light intensity was calibrated by a NREL‐certified standard Si solar cell with KG‐5 filter (SRC‐2020, Enlitech). The device active area via the aperture shade mask was estimated to be 0.034 cm^2^. The EQE spectra from 300 to 850 nm were collected by a QE‐R system (Enlitech). The electroluminescence (EL) spectra were recorded by a Kymera‐328I spectrograph and a Si EMCCD camera (DU970PBVF, Andor). EL efficiency (EQE_EL_) was carried out via a digital source meter (Keithley 2400) for injecting current into perovskite solar cells and a picometer (Keithley 6482) with a Si photodiode for quantifying photons emitted from the devices.

## Conflict of Interest

The authors declare no conflict of interest.

## Supporting information



Supporting Information

## Data Availability

The data that support the findings of this study are available from the corresponding author upon reasonable request.
