# Supplementary material for: Unveiling Charge Transfer and Recombination Dynamics in 3D/2D Heterostructure via Ultrafast Spectroscopy for Efficient Perovskite Solar Cells
Source: Adv Sci (Weinh). 2025 Jul 10;12(36):e08123. doi: 10.1002/advs.202508123 (PMC12462917; doi:10.1002/advs.202508123)
Supplement: Supplementary file 1 — Supporting Information [file ADVS-12-e08123-s001.docx]

Supporting Information

**Unveiling Charge Transfer and Recombination Dynamics in 3D/2D Heterostructure via Ultrafast Spectroscopy for Efficient Perovskite Solar Cells**

*Di Li†, Junhan Xie†, Shaobing Xiong, Xiaoxiao Zang, Zhennan Lin, Yuning Wu, Weimin Liu*, Bo Li*, Zhenrong Sun, Junhao Chu, Qinye Bao**

D. Li, S. Xiong, X. Zang, Z. Lin, Prof. Y. Wu, Prof. B. Li, Prof. Z. Sun, Prof. Q. Bao

School of Physics and Electronic Science, Engineering Research Center for Nanophotonics and Advanced Instrument (MOE), East China Normal University, Shanghai 200241, China

E-mail: bli@ee.ecnu.edu.cn; qybao@clpm.ecnu.edu.cn

J. Xie, Prof. W. Liu

School of Physical Science and Technology, ShanghaiTech University, Shanghai 201210, China

E-mail: liuwm@shanghaitech.edu.cn

Prof. J. Chu

Shanghai Frontiers Science Research Base of Intelligent Optoelectronics and Perception, Institute of Optoelectronics, Fudan University, Shanghai 200433, China

Prof. Q. Bao

Collaborative Innovation Center of Extreme Optics, Shanxi University, Taiyuan, Shanxi 030006, China

†These authors contributed equally to this work.

Keywords: 3D/2D perovskite heterostructure, charge transfer dynamics, nonradiative recombination, perovskite solar cell

**Supporting Note 1**

The carrier population depends on the diffusion and recombination processes of photogenerated carriers, and the TAM profiles are modeled as per the following theoretical framework:^[1-2]^ $\frac{\partial n\left( x, y,t \right)}{\partial t}=D\left( \frac{\partial^{2}n\left( x, y,t \right)}{\partial x^{2}}+\frac{\partial^{2}n\left( x, y,t \right)}{\partial y^{2}} \right)-k_{1}n\left( x,y,t \right)$, where *n*(*x*, *y*, *t*) is carrier population as a function of position and delay time, *D* is intrinsic spatial diffusion constant,^[3]^ *k*_1_ is monomolecular recombination coefficient.^[4]^ Here, the carrier population is described by the two-dimensional Gaussian function $n\left( x, y,t \right)=Nexp\left( -\frac{\left( x-x_{0} \right)^{2}}{2\sigma_{x,t}^{2}}-\frac{\left( y-y_{0} \right)^{2}}{2\sigma_{y,t}^{2}} \right)$, where $\sigma_{x,t}^{2}$ and $\sigma_{y,t}^{2}$ are time-dependent FWHM of Gaussian profiles. The two-dimensional Gaussian function can be simplified to one-dimensional ($\sigma_{t}^{2}$) because the carrier process is isotropic, and $\sigma_{t}^{2}$ satisfies the linear formula $\sigma_{t}^{2}-\sigma_{0}^{2}=4Dt$ under low excitation density.^[5]^

**Figure S1.** Steady-state PL spectra of bare 3D on glass with different pump fluences.

**Figure S2.** Steady-state PL spectra of 3D/2D_L_ heterostructure on glass with different pump fluences.

**Figure S3.** Steady-state PL spectra of 3D/2D_S_ heterostructure on glass with different pump fluences.

**Figure S4.** Differential decay time as a function of delay time for bare 3D on glass. The dashed line represents *τ*_SRH_ lifetime, consistent with that calculated result by the quadratic equation.

**Figure S5.** Differential decay time as a function of delay time for 3D/2D_L_ heterostructure on glass. The dashed line represents *τ*_SRH_ lifetime, consistent with that calculated result by the quadratic equation.

**Figure S6.** Differential decay time as a function of delay time for 3D/2D_S_ heterostructure on glass. The dashed line represents *τ*_SRH_ lifetime, consistent with that calculated result by the quadratic equation.

**Figure S7.** (a) *J*-*V* curves of n-i-p PSCs employing 3D/2D_S_ heterostructures with different concentrations of 2D_S_ solution.

**Figure S8.** *J*-*V* curves obtained in forward and reverse scans of n-i-p PSCs employing 3D/2D_L_ heterostructures.

**Figure S9.** *J*-*V* curves obtained in forward and reverse scans of n-i-p PSCs employing 3D/2D_S_ heterostructures.

**Figure S10.** EQE spectra and integrated *J*_sc_ curves of the PSCs with 3D/2D_S_ and 3D/2D_L_ heterostructures.

**Figure S11.** Dependence of *J*_sc_ on light intensity for the devices with 3D/2D_L_ and 3D/2D_S_ heterostructures.

**Table S1.** The fitted lifetime of interfacial hole transfer *τ*_HT_, Auger recombination *τ*_Auger_ and a long infinite component *τ*_long_ in bare 3D, 3D/2D_L_ and 3D/2D_S_ heterostructures.

|  | *A*_HT_ (%) | *τ*_HT_ (ps) | *A*_Auger_ (%) | *τ*_Auger_ (ns) | *τ*_long_ |
| --- | --- | --- | --- | --- | --- |
| 3D |  | - | 100 | 1.83 | infinite |
| 3D/2D_L_ | 46.5 | 178 | 53.5 | 1.88 | infinite |
| 3D/2D_S_ | 47.5 | 159 | 52.5 | 1.81 | infinite |

**Table S2.** The fitted lifetime of interfacial electron transfer $\tau_{\mathrm{ET}}'$, interfacial hole transfer $\tau_{\mathrm{HT}}'$, Auger recombination $\tau_{\mathrm{Auger}}'$ and a long infinite component $\tau_{\mathrm{long}}'$ in SnO_2_/3D/2D_L_ and SnO_2_/3D/2D_S_ films.

|  | $A_{\mathrm{ET}}'$ (%) | $\tau_{\mathrm{ET}}'$ (ps) | $A_{\mathrm{HT}}'$ (%) | $\tau_{\mathrm{HT}}'$ (ps) | $A_{\mathrm{Auger}}'$ (%) | $\tau_{\mathrm{Auger}}'$ (ns) | $\tau_{\mathrm{long}}'$ |
| --- | --- | --- | --- | --- | --- | --- | --- |
| SnO_2_/3D/2D_L_ | 12.4 | 42.8 | 41.6 | 189 | 46.0 | 1.86 | infinite |
| SnO_2_/3D/2D_S_ | 13.5 | 25.7 | 43.8 | 166 | 42.7 | 1.82 | infinite |

**Table S3.** Literature survey of 3D/2D heterostructure MAPbI_3_ PSCs.

| Device architecture | *V*_oc_  (V) | *J*_sc_  (mA cm^-2^) | FF  (%) | PCE  (%) | Ref. |
| --- | --- | --- | --- | --- | --- |
| ITO/PTAA/MAPbI_3_/(BA)_2_PbI_4_/PCBM/C_60_/BCP/Cu | 1.110 | 22.49 | 78 | 19.56 | ^[6]^ |
| ITO/MoO_3_/MAPbI_3_/PEA_2_Pb_2_I_4_/C60/BCP/Cu | 1.061 | 21.8 | 76.6 | 17.7 | ^[7]^ |
| FTO/SnO_2_/MAPbI_3_/DMePDAI_2_/spiro-OMeTAD /Au | 1.131 | 23.19 | 79.7 | 20.9 | ^[8]^ |
| ITO/PTAA/(PPA)_x_(MAPbI_3_)_1-x_/MAPbI_3_/BCP/Al | 1.06 | 25.92 | 80 | 21.98 | ^[9]^ |
| FTO/PTAA/MAPbI_3_/TPA_2_PbI_4_/doped PTAA/Ag | 1.130 | 23.6 | 82.5 | 22.00 | ^[10]^ |
| ITO/PTAA/PEA_2_PbI_4_/MAPbI_3_/C_60_/BCP/Al | 1.080 | 24.39 | 84 | 22.13 | ^[11]^ |
| FTO/TiO_2_/SnO_2_/MAPbI_3_:PEA_2_PbI_4_/PEA_2_PbI_4_/Spiro-OMeTAD /Ag | 1.136 | 23.57 | 82.7 | 22.14 | ^[12]^ |
| ITO/SnO_2_/MAPbI_3_/BA_2_MA_2_Pb_3_I_10_/Spiro-OMeTAD/MoO_3_/Ag | 1.151 | 24.35 | 79.45 | 22.32 | **This work** |

References:

[1] S. Deng, E. Shi, L. Yuan, L. Jin, L. Dou, L. Huang, *Nat. Commun.* **2020**, 11, 664.

[2] Z. Guo, Y. Wan, M. Yang, J. Snaider, K. Zhu, L. Huang, *Science* **2017**, 356, 59.

[3] J. Xie, W. Zhou, H. Li, Z. Wang, J. Jiang, Y. Zhang, X. Shen, Z. Ning, W. Liu, *Adv. Optical Mater.* **2024**, 12, 2303004.

[4] E. Menéndez-Proupin, P. Palacios, P. Wahnón, J. C. Conesa, *Phys. Rev. B* **2014**, 90, 045207.

[5] Z. Guo, J. S. Manser, Y. Wan, P. V. Kamat, L. Huang, *Nat. Commun.* **2015**, 6, 7471.

[6] Y. Lin, Y. Bai, Y. Fang, Z. Chen, S. Yang, X. Zheng, S. Tang, Y. Liu, J. Zhao, J. Huang, *J. Phys. Chem. Lett.* **2018**, 9, 654.

[7] M.-G. La-Placa, L. Gil-Escrig, D. Guo, F. Palazon, T. J. Savenije, M. Sessolo, H. J. Bolink, *ACS Energy Lett.* **2019**, 4, 2893.

[8] F. Zhang, S. Y. Park, C. Yao, H. Lu, S. P. Dunfield, C. Xiao, S. Uličná, X. Zhao, L. Du Hill, X. Chen, X. Wang, L. E. Mundt, K. H. Stone, L. T. Schelhas, G. Teeter, S. Parkin, E. L. Ratcliff, Y.-L. Loo, J. J. Berry, M. C. Beard, Y. Yan, B. W. Larson, K. Zhu, *Science* **2022**, 375, 71.

[9] T. Zhu, L. Shen, H. Chen, Y. Yang, L. Zheng, R. Chen, J. Zheng, J. Wang, X. Gong, *J. Mater. Chem. A,* **2021**, 9, 21910.

[10] Y. Wang, B. Li, H. Wang, Z. Zhang, Z. Dang, Y. Miao, K. Ma, Z. Qin, L. Lu, N. Zhang, Y. Wang, Y. Chen, Y. Zhao, *Adv. Mater.* **2025**, 37, 2419750.

[11] R. Chen, L. Shen, L. Zheng, T. Zhu, Y. Liu, L. Liu, J. Zheng, X. Gong, *ACS Appl. Mater. Interfaces* **2021**, 13, 49104.

[12] N. Wei, Y. Chen, X. Wang, Y. Miao, Z. Qin, X. Liu, H. Wei, Y. Zhao, *Adv. Funct. Mater.* **2021**, 32, 2108944.
